# The Ominous Aspergillus With Cancer of Blood Vessels: A Case of Invasive Aspergillosis and Epithelioid Angiosarcoma of the Lung

**DOI:** 10.7759/cureus.40034

**Published:** 2023-06-06

**Authors:** Sambhawana Bhandari, Maun R Baral, Mehndi Dandwani, Fnu Sandeep, Abhijith Hegde

**Affiliations:** 1 Internal Medicine, Danbury Hospital/Nuvance Health, Danbury, USA; 2 Pathology, Danbury Hospital/Nuvance Health, Danbury, USA; 3 Pulmonary and Critical Care Medicine, Danbury Hospital/Nuvance Health, Danbury, USA

**Keywords:** immunosuppression, invasive aspergillosis, pulmonary epithelioid angiosarcoma, aspergillosis, invasive pulmonary aspergillosis

## Abstract

Invasive aspergillosis occurs in the setting of risk factors such as severe or prolonged neutropenia, defects in cell-mediated immunity, and receipt of immunosuppressive therapy, particularly in patients with graft-versus-host disease (GVHD). Pulmonary epithelioid angiosarcomas (EASs) are rare malignant vascular tumors that are aggressive, frequently metastatic, and associated with a poor prognosis. We describe these two rare conditions occurring concurrently.

## Introduction

Aspergillosis refers to a spectrum of illnesses caused by *Aspergillus* species, ranging from a hypersensitivity reaction to invasive disease with significant mortality [1,2]. *Aspergillus* is a ubiquitous saprophytic mold commonly found in soil, water, and decaying food material. Invasive aspergillosis occurs in the setting of risk factors such as severe or prolonged neutropenia, defects in cell-mediated immunity, and receipt of immunosuppressive therapy, particularly in patients with graft-versus-host disease (GVHD) [3]. Pulmonary epithelioid angiosarcomas (EASs) are rare malignant vascular tumors that are aggressive, frequently metastatic, and associated with a poor prognosis. We describe two rare conditions occurring concurrently: invasive aspergillosis and epithelioid angiosarcoma.

## Case presentation

We present the case of a 55-year-old male who presented with complaints of exertional dyspnea and intermittent hemoptysis. He had a medical history of systemic lupus erythematosus (SLE) complicated by lupus nephritis necessitating a renal transplant (on immunosuppressive therapy with prednisone 5 mg, mycophenolate mofetil 720 mg twice daily, and tacrolimus 6 mg twice daily). Vital signs on presentation were as follows: temperature of 37.5°C, tachycardia at 110 beats/minute, respiratory rate of 20/minute, blood pressure of 110/70 mmHg, and oxygen saturation of 98% on room air. Blood work revealed a white blood cell (WBC) count of 6.9 × 10^9^/L, hemoglobin of 6.1 g/dL, hematocrit of 19.9%, mean corpuscular volume (MCV) of 94.8 fL, reticulocyte production index of 1.4, platelets of 181 × 10^9^/L, sodium of 134 mmol/L, potassium of 4.9 mmol/L, blood urea nitrogen (BUN) of 59 mg/dL, creatinine of 2.36 mg/dL, normal liver function test, peripheral blood smear showing normocytic normochromic anemia, mild anisocytosis, and teardrop cells (Table 1). Further workup with an iron panel was suggestive of chronic inflammation, and vitamin B12 levels were within normal limits. A direct Coombs test was negative (Table 1). Then, he underwent a colonoscopy that did not identify a source of bleeding. Bone marrow aspiration was negative for abnormal myeloid maturation, increased blast population, or lymphoproliferative disorder. Further studies including tests for paroxysmal nocturnal hemoglobinuria and celiac disease (tissue transglutaminase antibody) were negative.

**Table 1:** Laboratory values WBC: white blood cell, MCV: mean corpuscular volume, BUN: blood urea nitrogen

Laboratory tests	Results
WBC count	6.9 × 10^9^/L
Hemoglobin	6.1 g/dL
Hematocrit	19.9%
MCV	94.8 fL
Reticulocyte production index	1.4
Platelets	181 × 10^9^/L
Sodium	134 mmol/L
Potassium	4.9 mmol/L
BUN	59 mg/dL
Creatinine	2.36 mg/dL
Direct Coombs test	Negative

**Figure 1:**
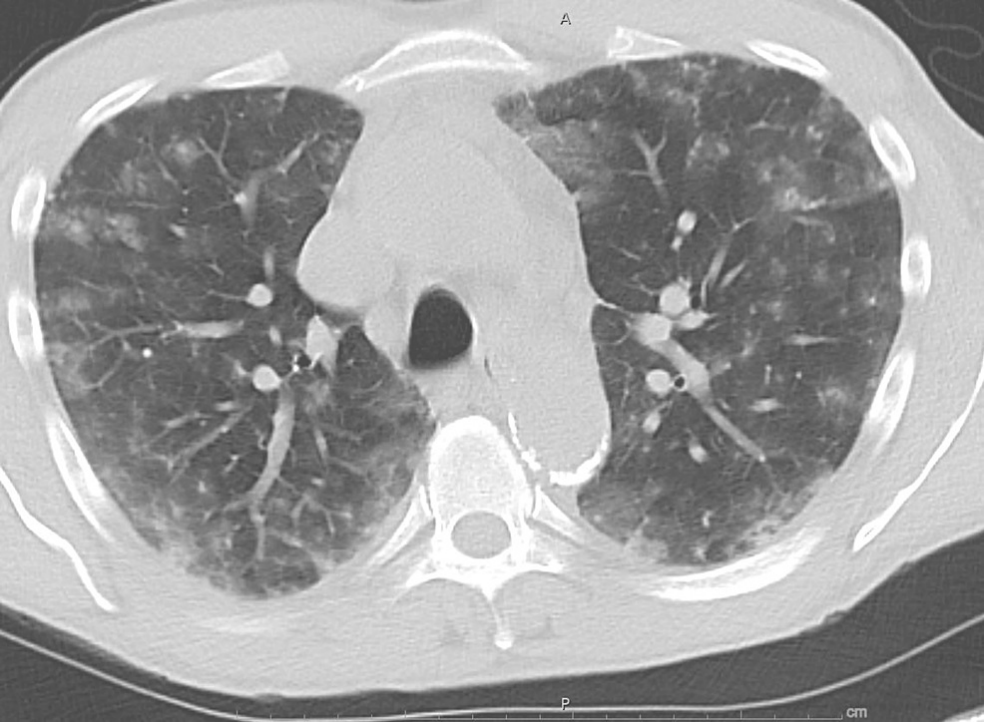
CT scan of the chest without contrast showing nonspecific diffuse ill-defined pulmonary nodules and ground-glass opacities CT: computed tomography

His hospital course was complicated by hypoxia, prompting a computed tomography (CT) scan of the chest, which showed diffuse ill-defined nodules and ground-glass opacities bilaterally (Figure 1). He was started on empiric antibiotics and underwent bronchoscopy with bronchoalveolar lavage, which showed diffuse infiltrates with blood throughout the tracheobronchial tree without an identifiable bleeding site suggestive of diffuse alveolar hemorrhage (DAH). Gram stain along with the stain for *Pneumocystis jiroveci* (PJP) was negative. An extensive autoimmune and infectious workup including antinuclear antibody (ANA) with reflex, cytomegalovirus (CMV) PCR, and beta-D-glucan was negative.

**Figure 2:**
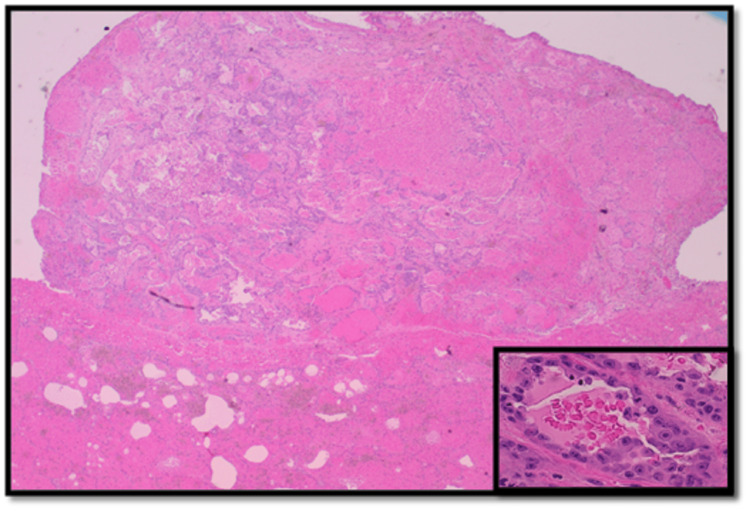
Sections show nodular growth of the lung composed of variably sized vascular spaces filled with extravasated blood (H&E, ×40), and higher magnification shows the vascular spaces lined by malignant epithelioid endothelial cells with large vesicular nuclei and prominent nucleoli (H&E, ×400) H&E: hematoxylin and eosin stain

**Figure 3:**
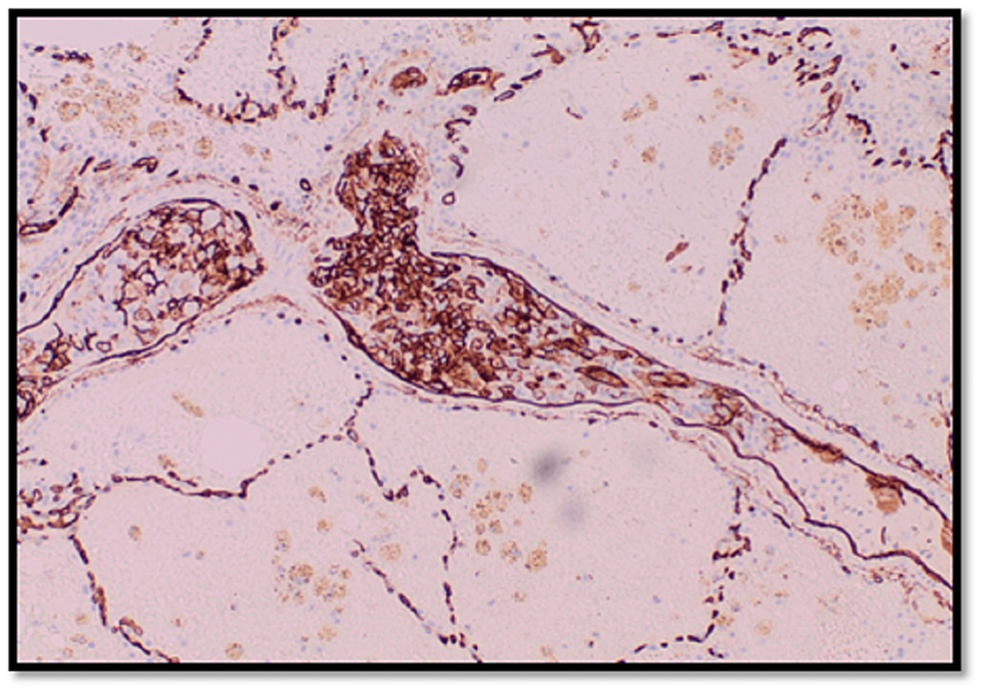
Section shows two vessels with tumor emboli; the tumor cells are highlighted by CD34 stain (IHC, ×100) CD34: cluster of differentiation 34, IHC: immunohistochemistry

**Figure 4:**
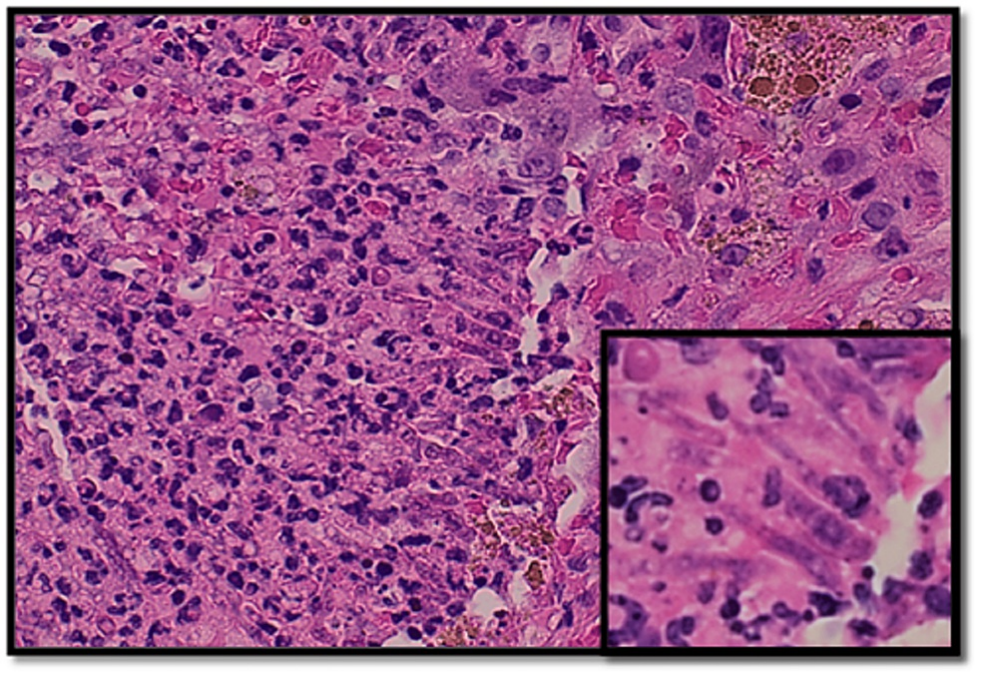
Section of the lung shows a focus with neutrophilic aggregates admixed with some fungal hyphae (H&E, ×200 and ×600) H&E: hematoxylin and eosin stain

**Figure 5:**
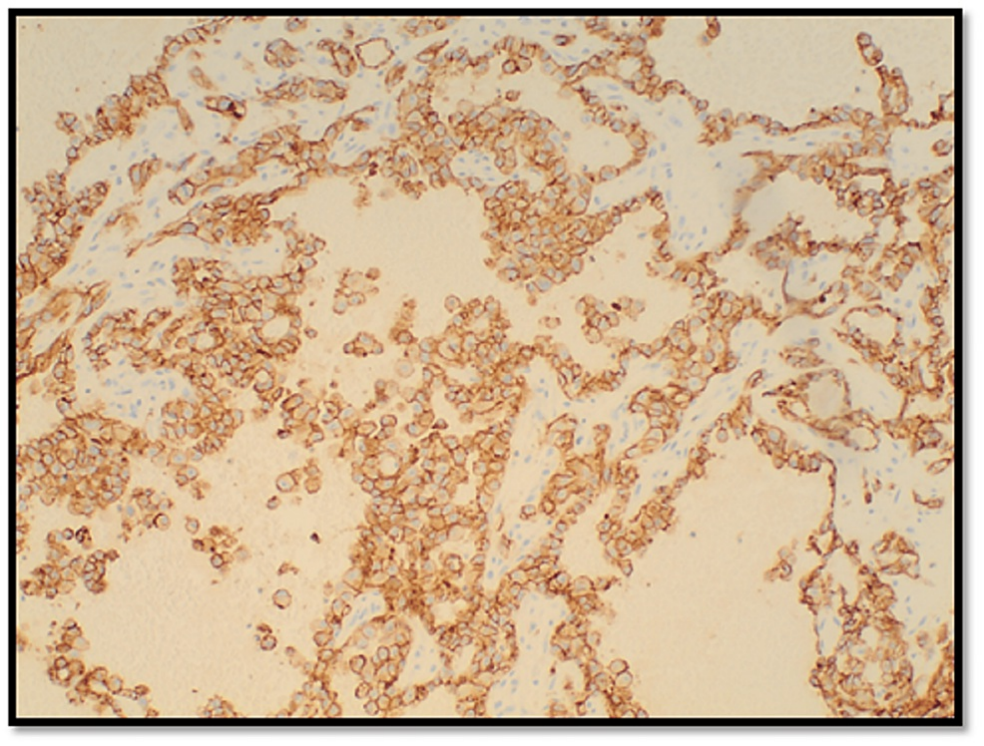
Section of the lesion shows vascular spaces lined by malignant epithelioid endothelial cells highlighted by CD31 stain (IHC, ×100) CD31: cluster of differentiation 31, IHC: immunohistochemistry

Ultimately, video-assisted thoracoscopic surgery (VATS)-assisted lung wedge biopsy was performed, which showed numerous microvascular tumor emboli as well as parenchymal and visceral pleural nodules composed of large epithelioid tumor cells arranged in nests, cords, and complex anastomosing vascular channels. There was marked nuclear pleomorphism with high mitotic activity and focal tumor necrosis. Some of the microvascular tumor emboli were partially or completely replaced by dystrophic calcification. Immunophenotyping showed strong immunoreactivity of the tumor cells for cluster of differentiation 31 (CD31) and cytokeratin CAM 5.2, patchy cytoplasmic staining for cluster of differentiation 34 (CD34), and absence of staining for cytokeratin 7, cytokeratin 20, thyroid transcription factor-1 (TTF-1), and Napsin consistent with epithelioid angiosarcoma. Additionally, multiple foci of necrotizing bronchopneumonia with associated invasive septate hyphae with 45-degree angle branching, morphologically consistent with *Aspergillus* species, were seen (Figures 2-5). This was further confirmed with Grocott’s methenamine silver (GMS) stain. Voriconazole therapy was initiated at this time.

His hospitalization was further complicated by an episode of massive hemoptysis with respiratory compromise requiring intubation and worsening kidney injury necessitating dialysis. He also had hemodynamic instability requiring vasopressor support. Eventually, goals of care discussions were held, and the family decided to focus on comfort measures only without any life-prolonging measures. The patient passed shortly after the withdrawal of care.

## Discussion

Pulmonary aspergillosis is a constellation of diseases that result from a complex interplay between the *Aspergillus* species and the host immunity. As such, clinical syndromes range from a hypersensitivity reaction to *Aspergillus* species to an invasive disease with high mortality rates [1].

Allergic bronchopulmonary aspergillosis (ABPA) is a complex hypersensitivity reaction to *Aspergillus* colonization that presents with dyspnea, chronic productive cough, and wheezing, usually in the setting of poorly controlled asthma and cystic fibrosis [2]. Chronic pulmonary aspergillosis (CPA) is another clinical syndrome seen in patients with underlying structural lung disease/cavitary lesion, as seen in patients with a history of tuberculosis [1], lung cancer, bronchiectasis, bronchial cysts, and chronic obstructive pulmonary disease (COPD) [3]. The presentation can range from nodules to cavitary lesions (chronic cavitary pulmonary aspergillosis) with or without an aspergilloma (with the potential to cause hemoptysis) or concomitant pleural fibrosis [1].

Invasive pulmonary aspergillosis (IPA) implies the invasion of lung tissue by *Aspergillus* hyphae seen in histological specimens [1]. The causative agents are one of the four species of *Aspergillus*: *Aspergillus fumigatus*, *Aspergillus flavus*, *Aspergillus niger*, and *Aspergillus terreus* [4]. It occurs in the setting of risk factors such as severe or prolonged neutropenia, defects in cell-mediated immunity, and receipt of immunosuppressive therapy, particularly in patients with graft-versus-host disease (GVHD) [2]. The symptoms are nonspecific and include fever, cough, sputum production, and dyspnea, which are unresponsive to antibiotics. Hemoptysis, as seen in our patient, is also a finding, especially with cavitary lesions. It can range from mild to massive. Pleuritic chest pain can be seen in the setting of pulmonary infarcts due to vascular invasion. Hematogenous dissemination to other organs is also a possibility, and brain involvement might lead to further serious complications [2].

Diagnosing IPA is challenging due to nonspecific signs and symptoms [2]. The gold standard of diagnosis is tissue diagnosis through thoracoscopic or open lung biopsy, which is often challenging [5]. Sputum analysis is often nonspecific as it may suggest colonization in immunocompetent individuals. Imaging studies, including computed tomography (CT) scans, have nonspecific findings. The most common presentations are pulmonary nodules and the halo sign, which is a zone of low attenuation due to hemorrhage surrounding the pulmonary nodule, both of which are neither sensitive nor pathognomonic [5]. Laboratory studies including antigen testing (primarily galactomannan (polysaccharide of the fungal wall)) and serum polymerase chain reaction (PCR) are also commonly used modalities. The sensitivity of antigen testing correlates with the severity of the disease but might allow early detection of the disease [2,6]. Bronchoscopy and bronchoalveolar lavage yielding specimens for culture, polymerase chain reaction (PCR), and antigen testing are also known diagnostic modalities; however, the yield is inconsistent [2].

Given high mortality rates, treatment should be initiated as soon as possible when there is a suspicion of the disease. Voriconazole is considered the treatment of choice with amphotericin B being an alternative [1]. Echinocandin derivatives are second-line therapies. Treatment is often prolonged, lasting up to more than a year. Reversal of immunosuppression is essential before deciding to stop treatment in most cases.

Epithelioid angiosarcomas (EASs) are extremely rare malignancies of endothelial origin that mostly arise from the deep soft tissue of the extremities, adrenal gland, bone, and thyroid gland [7]. Males are more commonly affected in the seventh decade of their life. They often demonstrate early nodal and solid organ metastasis, especially to the lungs, bone, soft tissue, and skin [8] with poor prognosis.

The diagnosis of EAS entails predominant endothelial morphology, which entails a rounded, polygonal, or fusiform morphology and abnormal endothelial cells forming vascular sinusoids. They are infiltrative and do not have a capsule or a clear border. Immunohistochemical staining is helpful in the diagnosis, and the markers of endothelial cell origin, namely, CD31, CD34, and factor VIII, are often positive. Like most angiosarcomas, epithelioid angiosarcomas are also positive for vimentin. Other positive markers are pancytokeratin, Ki67, and MIB-1 [8].

There have been prior case reports describing DAH in patients with pulmonary epithelioid angiosarcomas [9]; however, there have been no reports of pulmonary epithelioid angiosarcoma and invasive aspergillosis as described here.

## Conclusions

In our case report, we present a unique association between two uncommon and serious conditions: invasive pulmonary aspergillosis and pulmonary epithelioid angiosarcoma. Invasive pulmonary aspergillosis is caused by *Aspergillus* species and carries a bleak prognosis. Similarly, pulmonary epithelioid angiosarcomas are aggressive malignancies originating from endothelial cells and have a poor prognosis. Our patient was receiving immunosuppressive therapy, which may have increased their susceptibility to invasive pulmonary aspergillosis. However, it remains uncertain whether the presence of pulmonary epithelioid angiosarcoma also contributed to the development of invasive aspergillosis.
